# FACS-Based Isolation, Propagation and Characterization of Mouse Embryonic Cardiomyocytes Based on VCAM-1 Surface Marker Expression

**DOI:** 10.1371/journal.pone.0082403

**Published:** 2013-12-30

**Authors:** Annica Pontén, Stuart Walsh, Daniela Malan, Xiaojie Xian, Susanne Schéele, Laura Tarnawski, Bernd K. Fleischmann, Stefan Jovinge

**Affiliations:** 1 Lund Strategic Center for Stem Cell Biology and Cell Therapy, Lund University, Lund, Sweden; 2 Institute of Physiology I, Life & Brain Center, University of Bonn, Bonn, Germany; 3 Department of Cardiology Scania University Hospital, Lund, Sweden; University of Milan, Italy

## Abstract

Purification of cardiomyocytes from the embryonic mouse heart, embryonic stem (ES) or induced pluripotent stem cells (iPS) is a challenging task and will require specific isolation procedures. Lately the significance of surface markers for the isolation of cardiac cell populations with fluorescence activated cell sorting (FACS) has been acknowledged, and the hunt for cardiac specific markers has intensified. As cardiomyocytes have traditionally been characterized by their expression of specific transcription factors and structural proteins, and not by specific surface markers, this constitutes a significant bottleneck. Lately, Flk-1, c-kit and the cellular prion protein have been reported to specify cardiac progenitors, however, no surface markers have so far been reported to specify a committed cardiomyocyte. Herein show for the first time, that embryonic cardiomyocytes can be isolated with 98% purity, based on their expression of vascular cell adhesion molecule-1 (VCAM-1). The FACS-isolated cells express phenotypic markers for embryonic committed cardiomyocytes but not cardiac progenitors. An important aspect of FACS is to provide viable cells with retention of functionality. We show that VCAM-1 positive cardiomyocytes can be isolated with 95% viability suitable for *in vitro* culture, functional assays or expression analysis. In patch-clamp experiments we provide evidence of functionally intact cardiomyocytes of both atrial and ventricular subtypes. This work establishes that cardiomyocytes can be isolated with a high degree of purity and viability through FACS, based on specific surface marker expression as has been done in the hematopoietic field for decades. Our FACS protocol represents a significant advance in which purified populations of cardiomyocytes may be isolated and utilized for downstream applications, such as purification of ES-cell derived cardiomyocytes.

## Introduction

The identification of cardiac progenitors and their subsequent commitment and differentiation towards a mature cardiomyocyte lineage has in part been de-lineated. This has included the identification of three different progenitor-derived sources of *in vivo* cardiomyocytes - the primary and secondary heart fields [1] and the proepicardium [2]. Having tools to isolate purified and viable populations of cardiomyocytes and cardiac progenitors at different developmental stages would be of great importance to the cardiac field. To date, there has been no definitive protein epitopes identified that exclusively label cardiomyocytes. This lack of identified cardiac-specific surface markers has forced researchers to rely on transgenic reporter mice, knockout strains or advanced retrospective clonal analysis [3]. This is in contrast to the hematopoietic field where the delineation of the developmental hierarchy has been thoroughly established based on specific surface markers, and where FACS-sorting (Fluorescence activated cell sorting) of distinct progenitors and cell populations has been established not only in research but in routine clinical practice [4].

The cell surface markers associated with the earliest commitment stages in heart development, such as Flk-1 and c-kit, have been proposed by several investigators [5]–[8]. However, there are few specific markers and no expression of these epitopes has been observed on mature cardiomyocytes. Antibodies that react with the cell surface of human or murine cardiomyocytes and progenitors would potentially facilitate the purification of transplantable cells from Embryonic Stem Cell (ESC) derived sources. Any ESC or *in vitro* therapeutic strategy is dependent on the development of monoclonal antibody panels against extracellular markers that will facilitate efficient cell separation from mixed populations of cultured cells.

Apart from cardiomyocytes, the heart also consists of other cell types, including fibroblasts, smooth muscle cells, endothelial cells and circulating leukocytes. All these cell types needs to be removed prior to enrichment, which constitutes a great challenge. Traditionally, cardiomyocytes have been enriched from heart cell suspensions through Percoll gradient separation [9], or by pre-plating whereby non-cardiac cells can be excluded by their capacity to attach faster to cell culture plastic [10]. Neither of these methods will yield a pure cardiomyocyte cell population. Genetically tagged cells in transgenic mice engineered to express a fluorescent protein controlled by a cardiac specific promoter [7], [11] represent models where a high level of purity can be achieved. However, reporter mice are also limited in use, and require time-consuming genetic modifications and background crossing to generate a strain with both tagged cardiomyocytes and specific genetic modifications together. In addition, a genetic approach would also not be applicable for clinical purposes.

In the hematopoietic field, the FACS-technique has been long established for sorting and purifying specific cell populations [4]. A similar approach for isolating embryonic cardiomyocytes is proposed in this work. In the developing mouse heart, expression of the surface protein Vascular Cell Adhesion molecule-1 (VCAM-1) has been described from as early as embryonic day 8.75 (E8.75) until E13.5 [12]. Originally, VCAM-1 was identified as a member of the immunoglobulin superfamily, expressed by activated endothelial cells as response to cytokines and enabling the attachment of leukocytes [13]. Since then, VCAM-1 is known for its function as an adhesion molecule and as a signal transducer of inflammatory stimuli [14]. Others have recently reported VCAM-1 as a specific marker for cardiomyocytes derived from human iPS-cells or human ES-cells [15], [16]. High cardiomyocyte purity was obtained by FACS or magnetic bead separation, using specific antibodies against VCAM-1. In this study, we show for the first time that mouse cardiomyocytes can be isolated from the embryonic heart by their expression of VCAM-1 with high purity, high viability and with retained functionality. We have investigated the localization of VCAM-1 expression in embryonic and adult mouse heart and tested the specificity of the VCAM-1 surface marker as a tool to isolate cardiomyocytes by FACS from heterogeneous cardiac cell preparations. In addition, we have demonstrated the viability and suitability of VCAM-1 isolated cardiomyocytes by *in vitro* culture including electrophysiological studies. Our work may pave the way for future clinical studies where cardiomyocytes of high viability and purity from genetically unaltered cell sources is a prerequisite.

## Materials and Methods

### Animals

Embryos and adult organs isolated from wild-type C57BL/6 (Taconic) or CD1 (Charles River) mice. All animal experiments were approved by the Local Swedish Animal Ethics Committee at Lund University and performed in accordance to the Directive 2010/63/EU of the European Parliament.

### Immunofluorescence staining

Adult mouse hearts or embryos were fixed at 4°C with Stefanini solution and equilibrated in 20% sucrose and cryo-sectioned (10 µm). Cultured cells were fixed 20 min on ice with 2% PFA. Sections were permeabilized in PBS/0.1% Triton X-100, blocked with 10% goat or donkey serum and stained with antibodies against cTropT, Nkx2.5, alpha-SMA, alpha-actinin, MEF2C, VCAM-1, PECAM-1, connexin 43 or GATA-4. A detailed description is outlined in **Data S1**.

### Tissue dissociation

Hearts from E9.5-E12.5 mouse embryos were dissociated into single cell suspension by several rounds of digestion in isolation buffer (concentrations in mmol/L): 130 NaCl, 5 KCl, 1.2 KH_2_PO_4_, 6 HEPES, 5 NaHCO_3_, 1 MgSO_4_, 5 Glucose, pH 7.5) supplemented with 0.03 mg/ml Liberase Blendzyme 3 (Roche), 0.4 mg/ml Collagenase B (Roche), or 0.125 mg/ml Collagenase IV (Sigma-Aldrich) supplemented with 0.36 mmol/L CaCl_2,_ at 37°C. Detached cells were resuspended in PBS/20% FCS and kept at +4°C with gentle agitation until staining.

### Flow cytometry

Embryonic heart cell suspensions were stained on ice in PBS containing 5% FCS using the following pre-titrated antibodies: rat anti VCAM-1 (10 µg/ml, BD Biosciences), mouse anti-rat PE (2 µg/ml, BD Biosciences), rat anti PECAM-1 -APC (0.5 µg/ml, BD Biosciences), or isotype controls rat-IgG2a and rat-IgG2a -APC at corresponding concentrations (BD Biosciences). Positive sorting gates for the VCAM-1 high positive and PECAM-1 negative cells were set according to unstained controls, isotype controls and single staining controls. To exclude non-viable cardiomyocytes, cells were also stained with the DNA binding dye To-pro-1 (Invitrogen) or 7-AAD (Sigma-Aldrich). Cell sorting was performed on a FACSAria (BD Biosciences) with 100 µM nozzle. Doublet cells were discriminated as previously described [17]. Viability of cardiomyocytes was assessed during every round of sorting as well as after sorting (aliquots of 100–200 of the sorted cells). In a thorough viability analysis, 50.000 sorted cells were stained with To-pro-1 and re-analyzed by FACS. The purity of cells gated for VCAM-1^+^ PECAM-1^−^ was also assesses after each isolation. Small aliquots of cells (100–200 of the sorted cells) were re-analyzed using the same settings for gating. The fraction of cells still within the positive sorting served as a quality control of cell purity. For cardiomyocyte purity analysis, FACS isolated cells were fixed with 2% PFA, permeabilized and stained in Perm/Wash solution (BD Biosciences) with mouse anti cTropT-FITC antibodies (9 µg/ml, Hytest Ltd), or isotype control antibodies at corresponding concentration (mouse IgG2b-FITC, BD Biosciences). Neonatal heart cell preparations served as positive control.

### Cell culture

Primary cultured cardiac fibroblasts (E17.5–E19.5) were irradiated (5000 rad), and frozen in aliquots. After purification by FACS, cardiomyocytes were collected in culture medium and seeded at 20.000–40.000 cells/cm^2^ on gelatin-coated (0.1%) chamber slides (Corning), or slides pre-coated with irradiated embryonic cardiac fibroblasts (50.000 cells/cm^2^). Cultures were maintained in DMEM supplemented with 20% FCS, Penicillin/Streptomycin and Glutamax (Invitrogen). The PKH67 membrane-labeling assay is described in the **Data S1**.

### BrdU incorporation assay

FACS-isolated cardiomyocytes were co-cultured with irradiated embryonic cardiac fibroblasts. After 4 days, BrdU (20 µM, Sigma) was added directly to the medium with a chase period of 24 h. Cells grown in absence of BrdU and irradiated fibroblasts served as negative controls. Fixed cells were permeabilized in PBS/0.1% Triton X-100 supplemented with 6 mM MgCl_2_ and 1 mM CaCl_2_ and pre-treated with DNaseI (0.025 U/µl, Qiagen) at 37°C for 30 min prior to immunofluorescence staining. For quantification, 100 cardiomyocytes from 6 fields of visions at 20× magnification were evaluated.

### Quantitative PCR

RNA from whole E10.5 embryos, heterogeneous cell preparations from E10.5–11.5 hearts and the FACS-isolated VCAM-1^+^ PECAM-1^−^ population was extracted using RNeasy Plus Micro Kit (Qiagen). cDNA was synthesized from 70 ng total RNA (SuperScript First strand, Invitrogen) and real-time quantitative PCR (40 cycles) was performed using an iQ5 Real-time PCR thermal cycler with iQ SYBR-Green supermix (Bio-Rad). Absence of contaminations was confirmed using non-template and non-RT controls. In each gene-specific PCR, three biological replicates from different FACS experiments were compared in triplicate. The relative expression of each gene was calculated according to the 2^−ΔΔC^T method [18]. Expression of the housekeeping gene *GAPDH* was used to normalize for variations in RNA input. The specific primers used for cDNA amplification are listed in the **Data S1.**

### Electrophysiology and Ca^2+^ imaging

Performed as previously published [19], [20], described in detail in the **Data S1.**

## Results

### The surface marker VCAM-1 is expressed in embryonic myocardium

**Figure 1:**
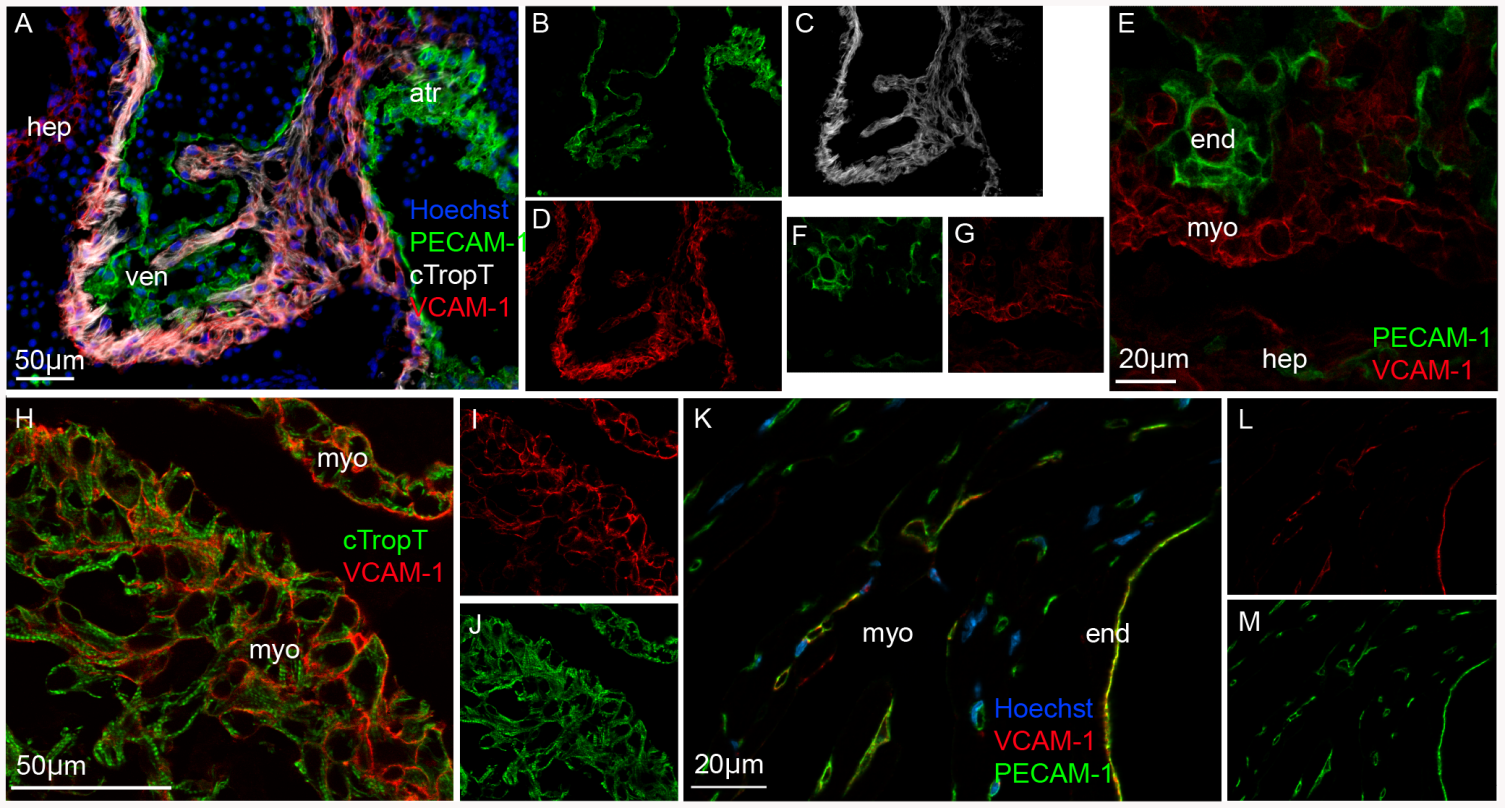
Expression of VCAM-1 surface marker in mouse embryonic myocardium. Immunofluorescence images of tissue cryo-sections showing that VCAM-1 is expressed by the myocardium in embryonic hearts (**A–J**) and by vascular endothelium in adult hearts (**K–M**). VCAM-1 co-localizes with cardiac specific cTropT (**A**) and is situated at the cell surface (**E, H**). VCAM-1 does not co-localize with endocardial/endothelial specific PECAM-1 in embryonic hearts (**A, E**). PECAM-1, VCAM-1 or cTropT in separate channels (**B–D, F–G, I–J and L–M**). Abbreviations: (atr) atria (hep) hepatic primordia (end) endocardium (myo) myocardium (ven) ventricle. Tissue sections: E10.5 (**A–D, H–J**); E12.5 (**E–G**); Adult (**K–M**). Optical sections: E–J.

We performed immunofluorescence staining of embryonic sections at E9.5, E10.5, E11.5, E12.5 and E14.5 (**Fig. 1**). As control, adult heart sections were analyzed. To specify myocardium we co-stained with antibodies against cardiac troponin T (cTropT). The onset of cTropT expression has been described to correlate with the onset of beating and cTropT is limited to the myocardial cells of the forming heart [21]. Since VCAM-1 expression has also been observed in activated endothelial cells [22], we also co-stained with antibodies against Platelet endothelial cell adhesion molecule-1 (PECAM-1). PECAM-1 is expressed by the endocardium and vascular endothelium in the developing heart but not by the myocardium [22].

At all embryonic stages examined, VCAM-1 staining was detected in the myocardium of both atria and ventricle, as shown by co-expression of cTropT (**Fig. 1A–D**). Confocal analysis demonstrated that VCAM-1 was present on the surface of cardiomyocytes (**Fig. 1 E–G** and **H–J**). We were unable to detect any expression of VCAM-1 in endocardial or vascular endothelial tissue with PECAM-1 antibody (**Fig. 1A–E**). At E14.5, VCAM-1 localization in the myocardium was diminished in comparison to earlier time points (data not shown). This observation confirms previous data which demonstrates that VCAM-1 expression is down-regulated after E13 [12]. In the adult heart, no expression of VCAM-1 was observed in the myocardium, instead, co-localization was observed with PECAM-1 in the endocardium, heart valves and vascular endothelium in keeping with previous reports [12] (**Fig. 1 K–M**). In summary, VCAM-1 specifies myocardium within the earlier stages of mouse heart development.

### VCAM-1 surface marker purification of embryonic cardiomyocytes by FACS

**Figure 2:**
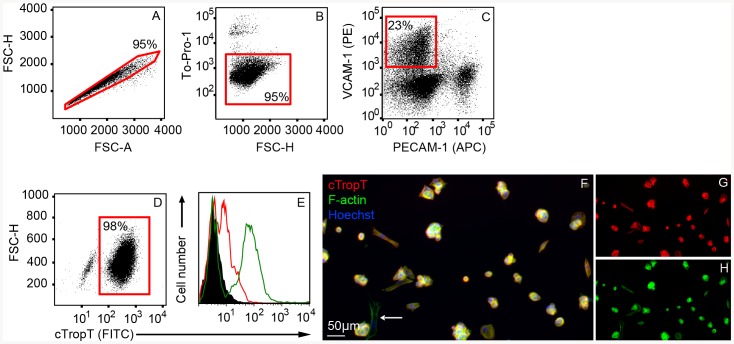
Purification of embryonic cardiomyocytes by Flow Cytometry. Flow Cytometry plots of E10.5–E11.5 cardiac cells labeled with specific antibodies to VCAM-1 and PECAM-1 (**A–C**). Cells are gated and sorted based on doublet discrimination (**A**), viability (**B**) and VCAM-1 positive PECAM-1 negative population (**C**). Flow Cytometry plot of sorted fixed cells, stained with cTropT antibodies to verify cardiac identity (**D**). Flow Cytometry histograms (overlays) showing control cTropT staining of neonatal hearts (**E**). Un-stained cells (black), isotype control (red outline) and neonatal heart cells (green outline). The percentage of gated cells through each step of the sort is indicated in each plot. Immunofluorescence staining (cTropT) of sorted cells cultured on gelatin coated slides for two days to verify cardiac identity and cell viability (**F**). F-actin and cTropT in separate channels (**G–H**). A low frequent cTropT-negative cell is indicated (arrow).

Single cell suspensions were obtained from E9.5–E12.5 hearts, stained with antibodies against VCAM-1 and PECAM-1, followed by FACS isolation (**Fig. 2**). Doublets (**Fig. 2A**) and non-viable cells (**Fig. 2B**) were excluded prior to FACS isolation of the VCAM-1 high positive and PECAM-1 negative cells (**Fig. 2C**). Although VCAM-1 and PECAM-1 expression did not show any co-localization in embryonic tissue cryo-sections, a low number of double positive cells were detected (<1% of total cells) in flow cytometric analyses and subsequently excluded during FACS-isolation.

To ensure the reproducibility of our FACS-based isolation strategy we analyzed cardiomyocytes isolated at different developmental stages, ranging from E9.5 to E12.5 (**Table S1**). We demonstrate that VCAM-1 can be utilized to purify cardiomyocytes from cardiac cell preparations at all stages included in the study (**Table S1**). The viability of cells during and post FACS isolation was very high (≥95%), regardless of developmental stage. The purity and cell yield was quite similar from hearts ranging from E9.5 to E11.5 embryos (**Table S1 and S2**). When E12.5-hearts were evaluated the sorting efficiency decreased (Table S1 and data not shown). This may be due to a reduced efficiency in the dissociation of larger hearts, leading to formation of cell aggregates (data not shown). To ensure a consistent purity for subsequent experiments, embryos at stages E10.5–E11.5 were used. E9.5-aged embryos were also included in this study. In a typical FACS isolation procedure, 95% of the cells were viable during the enrichment process (**Fig. 2B**) and 23% of the viable singlet cells were VCAM-1^+^ and PECAM-1^−^ (**Fig. 2C**). In a previous work using transgenic animals with cardiomyocyte specific GFP-expression, we have shown by Flow Cytometry that ∼30% of the total viable singlet cells in the embryonic heart are cardiomyocytes [23]. In this work, the yield was lower (∼20%). It is possible that high expression of VCAM-1 is very specific for distinct main populations of cardiomyocytes whereas other subpopulations of cardiomyocytes do not express VCAM-1. As shown by subsequent analysis described below,VCAM-1 positive cardiomyocyte represent cells of a more mature phenotype, and not immature cardiac progenitors.

The purity of isolated embryonic cardiomyocytes in terms of cTropT expression was evaluated by two independent methods. First, 50.000 cells from four individual isolations were fixed and stained with antibodies against cTropT, followed by re-analysis by FACS. We demonstrate that 98% of VCAM-1^+^ cells are also cTropT positive (**Fig. 2D** **and Table S2**). The specificity of the cTropT staining was verified using fixed cells from neonatal hearts (**Fig. 2E**). Heart cells stained for cTropT (green outline) results in a positive population (cardiomyocytes) and a negative population (non-cardiomyocytes). Second, the FACS-isolated cells were cultured on gelatin-coated slides and subjected to cTropT, F-actin, and Hoechst immunofluorescence staining (**Fig. 2 F–H** **and Table S1**). Of 2400 F-actin and Hoechst positive cells evaluated from two individual sorts, 97% were positive for the cardiomyocyte-specific structural marker cTropT.

In addition to the cTropT-reanalysis, a purity check was performed during every round of FACS-isolation. A small fraction of isolated cells were re-analyzed as a purity check of VCAM-1 expression. Of the FACS-isolated cardiomyocytes, 86–98% of the cells remained in theVCAM-1 positive PECAM-1 negative gate (Table S1). This level of purity was consistent between different isolations. This high degree of purity correlates with the data obtained from cTropT reanalysis. The small non-cardiomyocyte fraction of cells observed were found to be of a mesenchymal phenotype and further discussed in Data S1. In summary, these results demonstrate that FACS-based isolation using the VCAM-1 surface marker is an efficient method to obtain pure viable populations of cardiomyocytes.

### Cardiac cells expressing VCAM-1 exhibit a gene expression pattern characteristic of lineage committed embryonic cardiomyocytes

**Figure 3:**
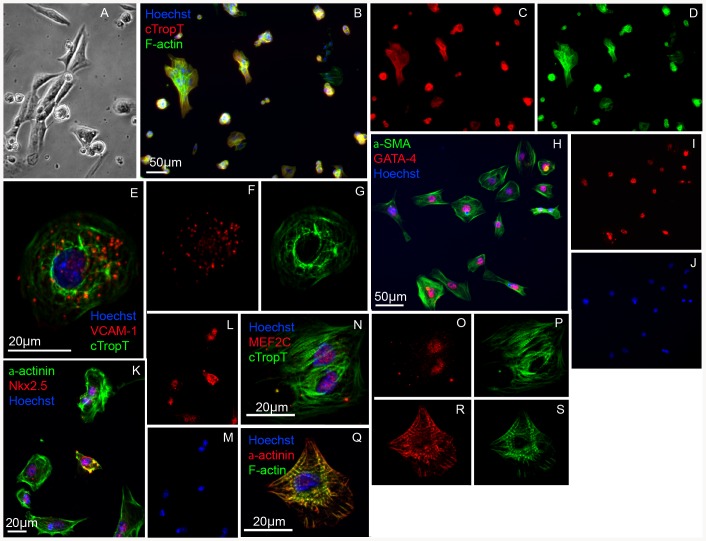
Protein expression profile of FACS-isolated VCAM-1^+^ embryonic cardiomyocytes. Phase contrast image of FACS-isolated cells grown on gelatin for 48 hours (**A**). Immunofluorescence images of the gelatin-cultured cells (**B–S**). Sorted cells positive for embryonic cardiac markers cTropT (**B–D**), αlpha-SMA and GATA4 (**H–J**) and Nkx2.5 and alpha-actinin (**K–M**). Confocal analysis verifying expression of VCAM-1 (**E–G**), expression of alpha-actinin (**Q–S**) and MEF2C (**N–P**).

To further characterize the VCAM-1 positive cardiac cell population, we transferred FACS-isolated cells to chamber slides coated with gelatin. Cellular attachment proceeded by beating cells, suggesting a high degree of intact functional cardiomyocytes (**Fig. 3A**), confirming our data from the FACS analysis (**Table S1**). After fixation, we investigated expression of phenotypic markers for cardiac cells. The results demonstrate that the cells express cardiac specific structural proteins cTropT (**Fig. 3B–G, N–P**) and alpha-actinin (**Fig. 3K–M and Q–S**), including alpha-SMA (**Fig. 3 H–J**) which is known to be expressed by embryonic but absent in adult cardiomyocytes [24]. Furthermore, we detected expression of the cardiac transcription factors Nkx2.5 (**Fig. 3K–M**), GATA-4 (**Fig. 3H–J**) and MEF2C (**Fig. 3N–P**). As a control we verified the presence of VCAM-1 (**Fig. 3 E–G**) as well as the absence of PECAM-1 (data not shown). We estimated that 100% of the FACS-isolated cells expressed alpha-SMA, 80% GATA-4 and 85% expressed Nkx2.5.

**Figure 4:**
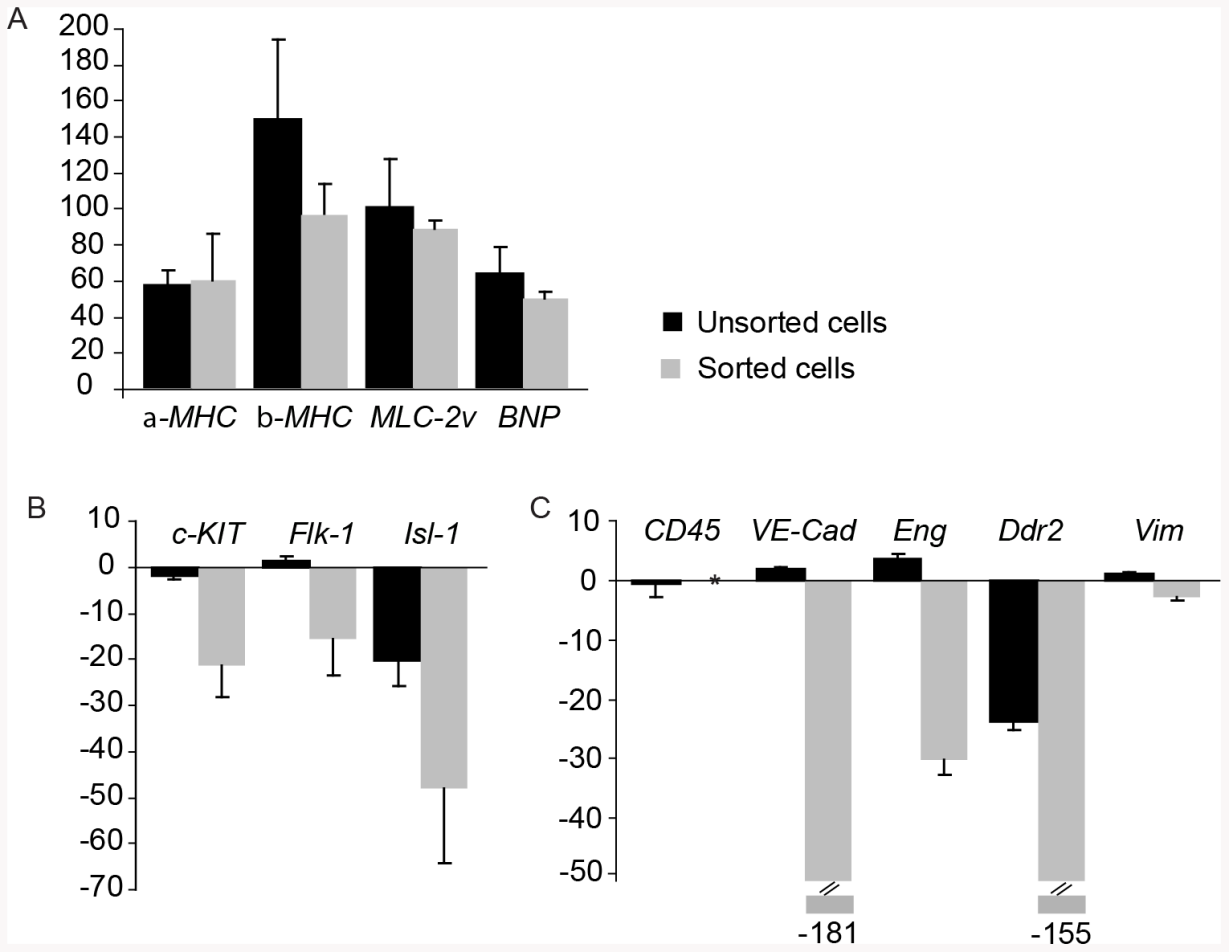
RNA expression profile of isolated VCAM-1^+^ embryonic cardiomyocytes. Gene expression, analyzed by real-time quantitative PCR, in E10.5–E11.5 un-sorted cardiac cells and sorted VCAM-1 positive cells compared to whole embryos. The fold change in gene expression is indicated by the Y-axis with standard deviation error-bars. Variations in RNA input were normalized through expression of the housekeeping gene *GAPDH*. Verification of cardiac-specific lineage genes alpha-*MHC*, *beta-MHC BNP* and *MLC-2v* (**A**). Differential expression of cardiac progenitor markers *c-KIT*, *Flk-1* and *Isl-1* (**B**) and gene markers for non-myocyte lineages, including hematopoietic (*CD45*), endothelial (*VE-Cadherin (VE-Cad)* and *Endoglin (Eng)*), fibroblasts (*Ddr2*) and mesenchymal cells (*Vimentin (Vim)*) (**C**). * Below detection level.

A thorough gene expression analysis further established the cardiac identity, purity and developmental profile of the VCAM-1 positive cells. RNA levels were measured in cardiac cells before and after FACS by real-time quantitative PCR, in three biological replicates (**Fig. 4**). We aimed to complement the protein expression analysis with RNA expression, in order to provide further evidence that VCAM-1 expression could specifically label cardiomyocytes, but not other cell types present in the heart. The relative expression for each gene was estimated by comparing each of the two samples with RNA from entire E10.5 embryos as reference. As positive controls, expression of cardiac specific genes were analysed: alpha-*MHC*, *beta-MHC*, *MLC-2v* and *BNP*. The results clearly showed that all genes were highly expressed both in the mixed cardiac cell population before FACS, as well as the sorted cells (**Fig. 4A**). The expression of progenitor and endothelial markers, *c-KIT* and *Flk-1*, was expressed by the unsorted cardiac cells, whereas the expression was down-regulated in the FACS-isolated cells (**Fig. 4B**). The absence of VCAM-1^+^ Flk-1^+^ and VCAM-1^+^ c-kit^+^ cells was also confirmed by immunofluorescence staining (**Fig. S1**) and by FACS analysis (**Fig. S2**). Finally, we investigated the expression of genes encoding markers for hematopoietic, endothelial, fibroblast and smooth muscle cells (**Fig. 4C**). The pan-hematopoietic marker *CD45* was detected in both whole embryo and un-sorted cardiac cells, confirming the presence of early hematopoietic cells at E10.5 and onwards [25], whereas the expression in the sorted population was below detection level. The absence of VCAM-1^+^ CD45^+^ cells was also confirmed by FACS analysis (**Fig. S2**). In the same analysis, we also show absence of the hematopoietic marker Sca-1 (**Fig. S2**). The endothelial cell markers *VE-Cadherin* and *Endoglin* were highly expressed in unsorted cardiac cells. This was expected since the embryonic heart contains a high portion of endocardial cells and newly formed blood vessels. In FACS-isolated populations however, the expression was heavily down-regulated, suggesting the absence of endothelial cells. The fibroblast marker *Ddr2* (Discoidin domain receptor family, member 2) was down-regulated many times in the sorted cells, suggesting the complete absence of fibroblasts. In the mixed cellular preparation from embryonic hearts, the down-regulation was less prominent, suggesting the presence of a low number of fibroblasts. At this early stage (E10.5–E11.5) fibroblast precursors are thought to be located in the epicardium, from where they start to colonize the heart [26]. We detected expression of *Vimentin*, a broad marker for mesenchymal cells, in all cell fractions: the un-sorted cells, the whole embryo and, to a lesser extent, also in the FACS purified cells. The expression of *Vimentin* most probably corresponds to the very small fraction of mesenchymal non-cardiomyocyte cells described above and in the **Data S1**.

Two other surface markers have also been suggested to specify early cardiomyocytes, SIRPA [27] and ALCAM (CD166) [28]. By FACS-analysis, outlined in **Data S1**, we were able to confirm a high degree of co-expression between VCAM-1 and SIRPA (85.4%) and to a lesser degree between VCAM-1 and ALCAM (51.1%) (**Fig. S4**). In summary, we show that the gene expression profile of sorted VCAM-1^+^ embryonic cardiomyocytes specifies a cell committed towards the cardiomyocyte lineage.

### Embryonic cardiomyocytes purified by FACS are intact and functional

**Figure 5:**
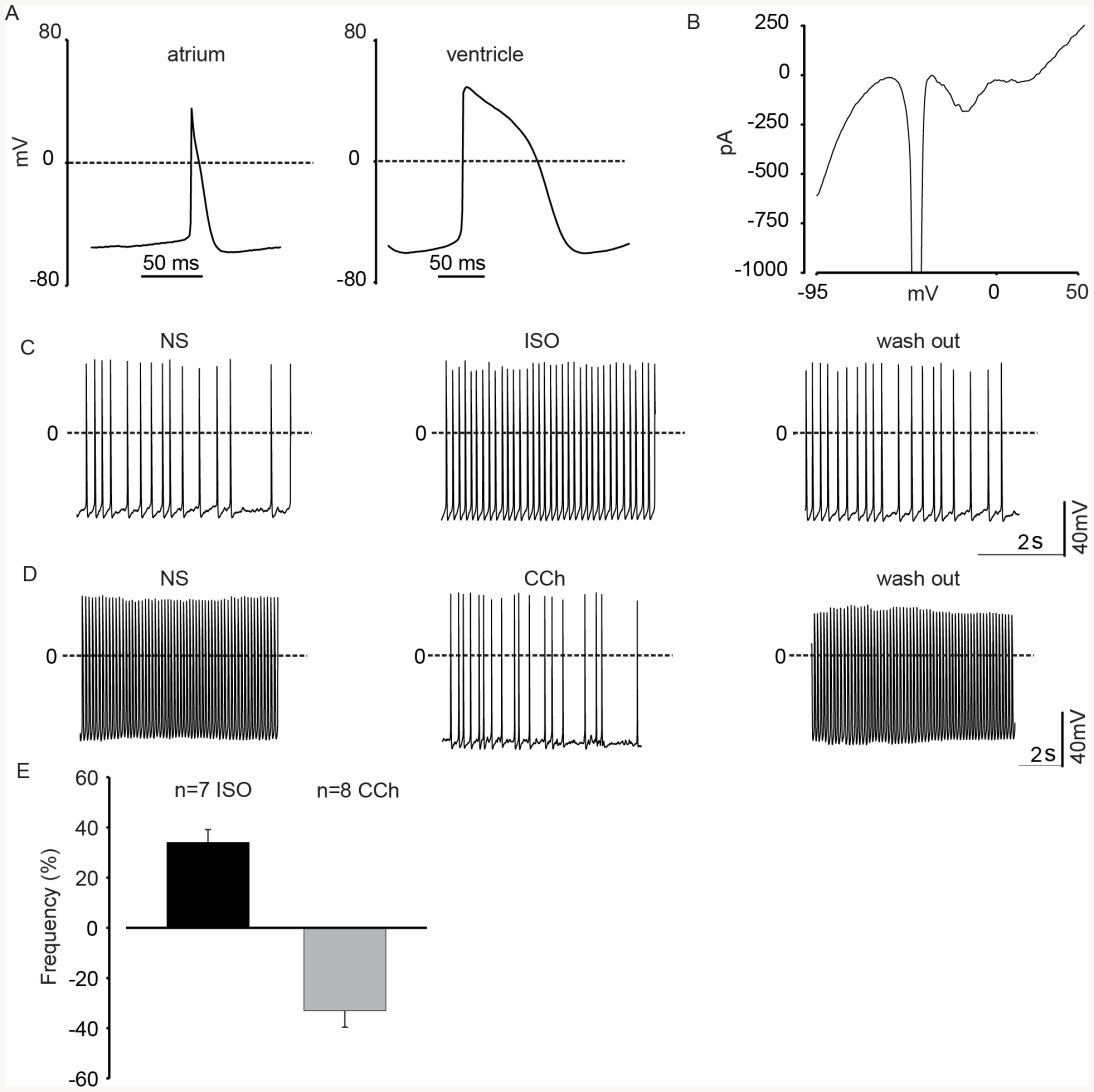
Functional analysis of embryonic cardiomyocytes purified by FACS. (**A**) Current clamp recordings revealed differentiation of FACS-isolated cells into cardiac subtypes: atrial and ventricle-like cells. (**B**) Representative voltage ramp protocol showing activation of inward and outward currents of a FACS-isolated cardiomyocyte. (**C**) APs recorded from a representative FACS-isolated cardiomyocyte, perfusion with the β-adrenergic agonist Isoprenalin evoked a positive chronotropic effect, this could be reversed upon wash-out. (**D**) APs recorded from a representative FACS-isolated cardiomyocyte, perfusion with the muscarinic agonist Carbachol induced a strong negative chronotropic effect, this could be reversed upon wash-out. (**E**) Statistics of the hormonal modulation of AP as % of frequency variation after the agonist application respect to the NS. Abbreviations: (NS) normal solution, (ISO) Isoprenalin, (CCh) Carbachol.

Our cell characterization methods clearly demonstrate efficient sorting of viable embryonic cardiomyocytes. However, in order to unequivocally demonstrate their cellular integrity and normal physiological function we performed patch clamp experiments. Based on action potential (AP) shape and duration we found two major cardiomyocyte subtypes, namely atrial- (n = 3, 16.6%) and ventricular (n = 13, 72.2%) -like cells (**Fig. 5A**); some cells (n = 2, 11.1%) possessed an undefined AP subtype. The key parameters of the APs were: maximum diastolic potential (MDP, atrial cells: −52.1±0.9 mV; ventricular cells: −64.8±3.5 mV), maximum rate of rise of the AP (dV/dt, atrial cells: 30.5±1.9 mV/ms; ventricular cells: 25.8±3.0 mV/ms), action potential duration at 50%(APD 50%), atrial cells: 24.3±4.7 ms; ventricular cells: 57.1±5.4 ms) and at 90% of repolarization (APD 90%), atrial cells: 38.4±6.6 ms; ventricular cells: 74.5±5.4 ms); as would be expected, MDP (unpaired t test p = 0.026) and APD90% (unpaired t test p = 0.014) differed significantly between the atrial- and ventricular-like cells. The key parameters of the APs were: maximum diastolic potential, atrial cells: −52.1±0.9 mV; ventricular cells: −64.8±3.5 mV), maximum rate of rise of the AP (dV/dt, atrial cells: 30.5±1.9 mV/ms; ventricular cells: 25.8±3.0 mV/ms), action potential duration at 50%(APD 50%), atrial cells: 24.3±4.7 ms; ventricular cells: 57.1±5.4 ms) and at 90% of repolarization (APD 90%), atrial cells: 38.4±6.6 ms; ventricular cells: 74.5±5.4 ms). These findings correlate well with our earlier electrophysiological studies on murine embryonic cardiomyocytes [29], [30]. Next, we looked at the functional expression of voltage dependent currents and could identify cardiomyocytes based on their voltage dependence I_Na_ (sodium current), I_CaT_ (calcium current, T-type), I_Ca, L_ (calcium current, L-type) as well as inwardly-and outwardly rectifying K^+^ currents (n = 14) (**Fig. 5B**).

To assess the integrity of signaling cascades we investigated the regulation of chronotropy by hormones of the autonomous nervous system. As depicted (**Fig. 5C, E**) the application of isoprenaline (1 µmol/L) resulted in a prominent increase in AP frequency of 44.2±8.6% (n = 9, paired t test p = 0.0002), whereas the acetylcholine analogue carbachol (CCh, 1 µmol/L) strongly decreased AP frequency, as previously reported [29], [31], in all cells by 33.0±6.5% (n = 8, paired t test p = 0.0012) (**Fig. 5D, E**). Moreover, in approximately one third of the cells tested, (n = 3 out of 8, 2 atrial-like and one undefined subtype) CCh induced a small hyperpolarization of the maximal diastolic potential by 4.5±0.1 mV, suggesting that these cells may be already further differentiated. Similar results as with CCh were also observed, when applying Adenosine (10 µmol/L), which led to a reduction of the beating frequency by 21.6±6.7% (n = 5, paired t test p = 0.043) (data not shown)..Besides the electrophysiology, we have also investigated the cytosolic Ca^2+^ ([Ca^2+^]_i_) homeostasis in spontaneously beating murine embryo-derived FACS-isolated VCAM^+^ PECAM^−^ cells. We could detect in all beating cardiomyocytes, (n = 51) typical [Ca^2+^]_i_ transients (**Fig. S5**). The upstroke of the action potential is at this stage of differentiation evoked by the opening of voltage dependent Na^+^ channels (see also above) and we therefore tested the effect of the Na^+^ channel blocker tetrodotoxin (TTX, 30 µM) on [Ca^2+^]_i_. As depicted in **Fig. S5A** application of TTX resulted in a blockade of the [Ca^2+^]_i_ transients (100%, n = 10), which is fully in line with the lack of action potentials in the presence of the drug; the TTX effect could be reversed upon wash-out.

We also tested the functional expression of ryanodine receptors using the agonist caffeine (10 mM). Our experiments revealed that application of caffeine evoked large [Ca^2+^]_i_ transients in most of the cells (95.1% of responding cells, n = 41) tested and that regular [Ca^2+^]_i_ transients re-appeared, once the intracellular stores were refilled (**Fig. S5B**).. Finally, we show that the calcium protein SERCA2ATPase is expressed by FACS-isolated cells. (**Fig. S3**). The calcium proteins are important for cardiac function. These data clearly show that PECAM^−^ VCAM^+^ cells display typical features of Ca^2+^ homeostasis for cardiomyocytes.

**Figure 6:**
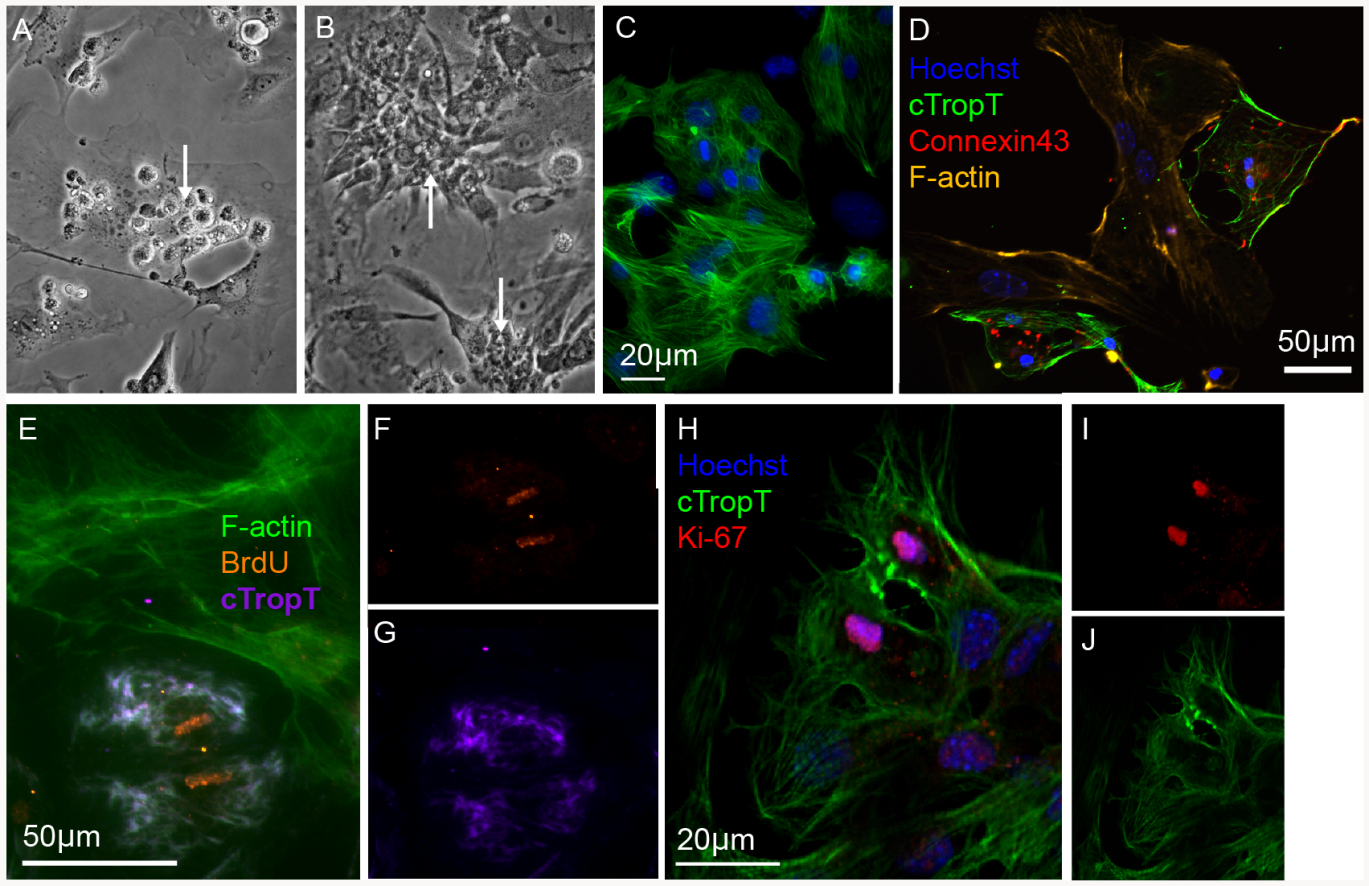
*In vitro* culture of primary embryonic cardiomyocytes after FACS. Phase contrast images of FACS-isolated cells grown on irradiated embryonic cardiac fibroblasts for two (**A**) or six (**B**) days. Rounded cells attached to the fibroblasts (**A**) and beating clusters of cardiomyocytes (**B, C**). Immunofluorescence images of the co-cultured cells (**C–J**). The FACS-isolated cells form a tight meshwork of beating cardiomyocytes in close contact with the surrounding fibroblasts as well as expressing cTropT (**C**) and Connexin43 (**D**). Co-cultures labeled with BrdU after five days for 24 h (**E–G**). Incorporation of BrdU is detected in cardiomyocytes but not in the surrounding irradiated fibroblasts. A optical section of a dividing cardiomyocyte in co-culture with fibroblasts for five days visualized by Ki-67 expression (**H–J**).

In addition to patch clamp experiments and Ca^2+^ imaging, we investigated the functionality of FACS-isolated cardiomyocytes in culture (**Fig. 6**). The cells were seeded on irradiated embryonic cardiac fibroblasts (**Fig. 6A**) and after several days were observed to be adherent to the fibroblast monolayer (**Fig. 6B**) forming clusters of cTropT positive cells (**Fig. 6C**) beating in synchrony (**Movie S1**), even in the absence of myocyte-to-myocyte contact. This observation confirms earlier studies showing that coupling with fibroblasts enables cardiomyocytes to beat in synchrony even over extended distances [32].

By immunofluorescence staining we could confirm that cardiomyocytes and fibroblasts were in contact and that the cells expressed gap junction protein Connexin43 (**Fig. 6D**) important for electrical coupling [33]. We have previously shown that embryonic cardiomyocytes have a strong capacity to proliferate, whereas neonatal or adult cells have a very limited capacity to do so [34].

To investigate if the FACS-isolated embryonic cardiomyocytes retain their capacity to proliferate *in vitro*, we examined cell proliferation by BrdU incorporation (**Fig. 6E–G** **and Table S1**) and by expression of the nuclear protein Ki-67 after 6 days *in vitro* (**Fig. 6H–J**). After a 24 h chase period, 84% of cardiomyocytes were BrdU-positive (**Table S2**). The results clearly demonstrate the presence of proliferating cardiomyocytes in co-culture with irradiated fibroblasts. Although BrdU incorporation and Ki-67 expression provide evidence of cell proliferation, those assays do not provide solid evidence of cell division. For this purpose, a PKH67 cell membrane labeling experiment was performed. In this assay, the cell membranes of live cells are labeled with a fluorescent cell linker dye. Whenever a cell produces two daughter cells, the fluorescence intensity will be approximately half. PKH67-labelled cardiomyocytes were analysed before and after 6 days in culture (**Fig. S6E–G**). As controls, irradiated embryonic cardiomyocytes were used in parallel (**Fig. S6A–D**). As expected, the irradiated fibroblasts did produce any daughter cells (**Fig. S6B and D**). The embryonic cardiomyocytes, however, showed an almost complete turnover of cells (>90%) towards new generations of daughter cells (**Fig. S6E–G**), as shown by the decline in fluorescence intensity. These results support our previous data on BrdU incorporation and presence of Ki-67 positive cells. Based on these results we conclude that our FACS-isolation protocol yields biologically and functionally intact embryonic cardiomyocytes. It does neither alter physiological function nor cellular signaling of embryonic cardiomyocytes.

## Discussion

VCAM-1 has previously been identified in the developing myocardium [12]. However, VCAM-1 is expressed in early organogenesis and thus expressed in several other organs during development. Herein we show that cardiomyocytes, which are among the first differentiated cells arising in organogenesis, can be readily identified by the VCAM-1 surface marker in early development. With the combined use of PECAM-1 (CD31) as a negative, and VCAM-1 as a positive surface-marker, we have demonstrated that primary cardiomyocytes can be efficiently isolated by FACS with a purity as high as 98%. This represents an important advance in the cardiac field for surface-marker based *in vivo* cardiomyocyte isolation, as has been done for decades in the hematopoietic field. In previous reports, embryonic stem (ES)-derived cells have been reported to express VCAM-1 in early cardiomyocyte differentiation, and accessible to cell sorting procedures [15], [16]. This may represent an important step towards a future cell-based therapy of the human heart. The maturity of differentiation and phenotype of these ES-derived or re-programmed cells *in vitro* compared to *in vivo* primary cardiomyocytes is however unclear. The development of a new source for the generation of cardiomyocytes by re-programming stresses the importance of having access to *in vivo* primary cardiomyocytes for comparison.

Our work is unique as we provide evidence that *in vivo*-derived primary cardiomyocytes can be efficiently isolated from the embryonic heart, using VCAM-1 expression as a tool. Beating cardiomyocytes have been identified as early as E.9.5 *in vivo*, even though there is a prominent change in cardiac morphology until E13.5 [12]. Studies on *in vivo* isolated cardiomyocytes from genetically modified mice would require a protease resistant surface marker for viable dissociation protocols. Such isolation protocols will always render the risk of either degrading surface markers or inducing them in other cells. We have tested alternative surface markers for the isolation of cardiomyocytes, as previously reported in the literature for human ES-derived cardiomyocytes [15], [16], [27], including SIRPA and CD166 ALCAM. In our hands, the *in vivo* primary cardiomyocytes exhibited a high degree of VCAM-1 co-expression with SIRPA, and to a lesser degree the CD166 antigen. CD166 is reported to be a cardiomyocyte antigen at earlier developmental time-points than assessed in this study, and it seems to loose its restriction to, and expression on cardiomyocytes earlier than VCAM-1 which could explain the lesser co-localization to VCAM-1^+^ than SIRPA antigen in the VCAM-1^+^/PECAM FACS isolated cells [28]. Previous reports of SIRPA expression in cardiac cells was observed on human cells [27], and we have shown expression in primary mouse embryonic cardiomyocytes.

In the field of cardiogenesis, several investigators have lately identified surface markers expressed by cardiac progenitors, including the cellular prion protein, c-kit and Flk-1 [6], [7]. These progenitor populations correspond to cells present during early cardiogenesis in the mouse embryo as well as differentiated ES-cells and are characterized by their capacity to develop into endothelial cells, smooth muscle cells and/or cardiomyocytes. Our work demonstrates that VCAM-1 does not select these cardiac progenitors but instead a population of cells further downstream in the cardiomyocyte developmental pathway.

We provide evidence that within the developing heart, VCAM-1 expression specifies cardiac cells from both atrial and ventricular compartments. Furthermore, we show that VCAM-1^+^ cardiomyocytes isolated by FACS remain viable, and retain their characteristic phenotype of a functional embryonic cardiomyocyte. Notably, the FACS-isolated cells proliferate in co-culture with fibroblasts. This observation is in agreement with our previous work showing that embryonic cardiomyocytes proliferate, whereas neonatal or adult cells have a very limited capacity to do so [34]. Thus, we provide the means to propagate these cells *in vitro*, which has always been challenging for mouse cardiomyocytes.

VCAM-1 works well together with our enzyme-based isolation protocol. Besides the very high purity of 98%, this protocol exceeds earlier published protocols in regard to performance and downstream characterization. In summary, we have developed a protocol for primary cardiomyocyte isolation that has taken many obstacles into account: Cell viability throughout tissue dissociation as well as FACS-isolation, cardiomyocyte purity, cell functionality in terms of intact electrophysiology as well as calcium homeostasis, and finally, a retained capacity to proliferate. For the first time we have established a method to isolate *in vivo* mouse primary embryonic cardiomyocytes by FACS, based on their specific expression of the surface marker VCAM-1. Previous reports on VCAM-1 or SIRPA-specific selection of cardiomyocytes have been performed using human ES-cells or iPS-cells [15], [16], [27]. Our protocol allows the isolation of highly viable and intact wild-type cells, suitable for any downstream application, including *in vitro* culture, electrophysiological studies, protein and RNA expression analysis or disease models studied *in vitro*.

## Supporting Information

Data S1 **A cardiomyocyte purity of 98% was achieved based on FACS isolation utilizing two surface-markers (VCAM-1^+^ and PECAM-1^−^).** We demonstrated that a small fraction of FACS-isolated cells were non-cardiomyocytes. We suggest that the remaining 2% non-cardiomyocytes are in fact mesenchymal cells originating from the atrioventricular canal and outflow tract, namely cardiac cushion cells. These cells co-express VCAM-1, α-SMA, CD34 and PDGFR-β but are negative for cTropT, GATA-4 and Flk-1 (**Fig. 1** **–** **2**). Cells co-expressing CD34, VCAM-1 and PDGFR-β were found in the in AV-canal cushion (**Fig. 1E and K**). The cushion cells are mesenchymal cells originating from epithelial-to-mesenchymal transition of the endocardium and will further differentiate into the valves and aortic arch vascular smooth muscle cells [35].
(DOC)

Figure S1 **Immuno-localization of VCAM-1 expressing non-cardiac mesenchymal cells.** Representative image of a small fraction VCAM-1+ sorted cells expressing α-SMA but not the cardiac marker GATA-4 (**A**). Immunofluorescence staining of embryonic tissue section showing expression of PDGFR-β in the epicardium, whereas VCAM-1 is expressed by the underlying myocardium (**B–D**). Optical sections showing co-localization of PDGFR-β with VCAM-1 in mesenchymal cells of the AV-canal cushion (**E–G**) but not with the surrounding cTropT-positive myocardium (**H–J**). Optical sections showing co-localization of CD34 with VCAM-1 in mesenchymal cells of the AV-canal cushion but not in endocardial or endothelial cells (**K–M**). Optical sections demonstrating no co-localization of Flk-1 with VCAM-1 in either myocardium, endocardium, or cushion cells (**N–P**). Abbreviations: (cush) AV canal cushion (end) endocardium (myo) myocardium (ven) ventricle. Tissue sections: E10.5 (**B–M**); E11.5 (**N–P**).
(TIF)

Figure S2 **FACS analysis of progenitor and stem cell markers in combination with VCAM-1.** Within the VCAM-1 positive population, 20% of the cells where CD34+ (**A**). No co-expressionwith the common leukocyte antigen CD45 (**B**) or the hematopoietic stem cell marker Sca-1(**C**) was detected. A very low fraction of VCAM-1+ cells expressed the progenitor markers Flk-1 or c-kit (**D, E**).
(TIF)

Figure S3 **Co-localization between calcium proteins and α-actinin on VCAM-1^+^/PECAM-1^−^ cells.** Immunofluorescence staining on FACS-isolated and cultured cells. Nuclear stain with DAPI (**A**), α-actinin (**B**) and SERCA2 ATPase (**C**) with all channels combined (**D**).
(TIF)

Figure S4 **Flow Cytometry analysis of the VCAM-1^+^/PECAM-1^−^ population and co-localization of SIRPA and ALCAM.** FSC-A and SSC-A gating of cells isolated from the heart (**A**). 89.4% of the cells were viable as assessed by 7-AAD (**B**). Isotype controls were performed for each antibody used (**C**, **E**). The VCAM-1^+^/PECAM-1^−^ population (**D**) is to a high degree (85.4%) positive for SIRPA (**F**) but to a lesser degree ALCAM^+^ (51.1%) (**F**).
(TIF)

Figure S5 **Ca^2+^ imaging of PECAM^−^ VCAM^+^ embryonic cardiomyocytes.** Spontaneously beating cardiomyocytes displayed regular [Ca^2+^]_i_ transients, which could be stopped by applying TTX (30 µM) (100%, n = 19), a blocker of voltage activated Na^+^ channels (**A**). When applying caffeine (10 mM), most of the cells (88.7%, n = 44) showed a large [Ca^2+^]_i_ transient (**B**) due to the emptying of the sarcoplasmatic reticulum stores, followed by a reduced amplitude of the [Ca^2+^]_i_ transients until refilling of the stores during wash-out.
(TIF)

Figure S6 **Cell division analysis of cultured embryonic cardiomyocytes by PKH67 labelling.** Flow Cytometry histograms showing fluorescence from PKH67-labelled cells before and after culture. Unstained (**A**) and PKH67-stained (**B**) irradiated embryonic fibroblasts before culture. Unstained (**C**) and PKH67-stained (**D**) irradiated embryonic fibroblasts after culture for 6 days. Increased cell autofluorescence is observed in unstained fibroblasts after culture (**C**) compared with before culture (**A**). No decay in fluorescence is observed from the PKH67-stained irradiated fibroblasts after culture (**D**) compared with stained cells before culture (**B**). Unstained (**E**) and PKH67-stained (**F**) FACS-isolated embryonic cardiomyocytes before culture. PKH76-stained cardiomyocytes (**G**) after co-culture with unstained irradiated embryonic fibroblasts for 6 days. Strong decay in fluorescence is observed from the PKH67-stained cardiomycoytes after culture (**G**) compared with stained cells before culture (**F**). As the stained cardiomyocytes are grown in co-culture with unstained irradiated fibroblasts, the histogram represents fluorescence from the PKH67-stained cardiomyocytes as well as autofluorescence from the irradiated fibroblasts. The dashed histograms represent fluorescence corresponding to unstained fibroblasts after culture (*, as in **C**) and fluorescence corresponding to stained cardiomyocytes before culture (**#**, as in **F**). **G0** denotes generation zero and the arrow denotes the presence of daughter generations.
(TIF)

Movie S1 **Seeded cardimyocytes on irradiated embryonic cardiac fibroblasts observed to be forming clusters of cTropT positive cells beating in synchrony.**
(AVI)

Table S1 **FACS statistics of VCAM-1^+^ PECAM^−^ cardiomyocytes.**
(DOC)

Table S2 **Quantitative analysis of FACS-isolated VCAM-1^+^ PECAM^−^ cardiomyocytes.**
(DOCX)
