# Supplementary material for: FACS-Based Isolation, Propagation and Characterization of Mouse Embryonic Cardiomyocytes Based on VCAM-1 Surface Marker Expression
Source: PLoS One. 2013 Dec 30;8(12):e82403. doi: 10.1371/journal.pone.0082403 (PMC3875414; doi:10.1371/journal.pone.0082403)
Supplement: Data S1 — A cardiomyocyte purity of 98% was achieved based on FACS isolation utilizing two surface-markers (VCAM-1+ and PECAM-1−). We demonstrated that a small fraction of FACS-isolated cells were non-cardiomyocytes. We suggest that the remaining 2% non-cardiomyocytes are in fact mesenchymal cells originating from the atrioventricular canal and outflow tract, namely cardiac cushion cells. These cells co-express VCAM-1, α-SMA, CD34 and PDGFR-β but are negative for cTropT, GATA-4 and Flk-1 ( Fig. 1 – 2 ). Cells co-expressing CD34, VCAM-1 and PDGFR-β were found in the in AV-canal cushion ( Fig. 1E and K ). The cushion cells are mesenchymal cells originating from epithelial-to-mesenchymal transition of the endocardium and will further differentiate into the valves and aortic arch vascular smooth muscle cells [35]. (DOC) [file pone.0082403.s001.doc]

**SUPPLEMENTARY METHODS**

**Immunofluorescence staining**

Adult hearts and embryos were fixed o/n at 4°C with Stefanini solution and further processed for cryo- sectioning (10 μm). Sections were permeabilized in PBS/0.1% Triton X-100, blocked with 10% goat or donkey serum (Sigma-Aldrich) and stained with primary antibodies: mouse anti cTropT (2.5μg/ml, Neomarkers), goat anti Nkx2.5 (1μg/ml, R&D systems), mouse anti α-SMA (2.5μg/ml, Sigma-Aldrich), rabbit anti MEF2C (1/200, antibody conc. not indicated, Cell Signaling), goat anti VCAM-1 (1μg/ml, R&D Systems), mouse anti α-actinin (2.5μg/ml, Sigma-Aldrich), rat anti PECAM-1 (1.25μg/ml, BD Biosciences), rabbit anti connexin 43 (2.5μg/ml, Alpha Diagnostics), goat anti GATA-4 (2μg/ml, Santa Cruz Biotechnology), rat anti PDGFR-β (1μg/ml, eBioscience), rat anti Flk-1 (2μg/ml, eBioscience), rat anti CD34 (2.5μg/ml, BD Biosciences), rat anti BrdU (1μg/ml, AbD Serotec), rabbit anti Ki-67 (1/1000, serum, Nova Castro), or anti-SERCA2 ATPase (1μg/ml Abcam). Primary antibodies were visualized with secondary antibodies conjugated to FITC/Cy3 (3.75μg/ml, Jackson ImmunoResearch) or Alexa Fluor 488/555/594/647 (5μg/ml, Invitrogen). Nuclei were stained with Hoechst 33342 or DAPI (Invitrogen) and F-actin was visualized with Phalloidin-Alexa Flour 488/555 (Invitrogen). Cultured cells were fixed 20 min on ice with 2% PFA before staining. Imaging was performed using Axiovert 200M fluorescence microscope equipped with apotome (ZEISS), a laser scanning confocal microscope TCS (LEICA), or a spinning disk confocal microscope (Olympus IX-81). In quantitative analysis, random fields at 10x magnification were selected for cell counting. For cTropT quantification, 2400 cells from two different sorts were counted and for α-SMA, GATA-4 and Nkx2.5 quantifications, approximately 100 cells were counted.

**FACS analysis of VCAM-1 expression in combination with other cardiac, hematopoietic or stem-cell markers.**

Hearts from E10.5-11 mouse embryos were dissociated into single cell suspension as described in materials and methods. VCAM-1 staining in combination with hematopoietic, endothelial, progenitor and stem cell markers, was performed using the following pre-titrated antibodies: rat anti VCAM-1 AF647 (4μg/ml, eBioscience), rat anti Flk-1 Pacific Blue (4μg/ml, eBioscience), rat anti CD45 PE (4μg/ml, BD Biosciences), rat anti c-kit eFlour780 (1μg/ml, eBioscience), rat anti CD34 FITC (8μg/ml, BD Biosciences) and Sca-1 FITC (0.8μg/ml, BD Biosciences). Positive gates were set according to FMO (Fluorescense Minus One) and unstained controls. As a positive control for all markers included, E14.5 liver cells were stained in parallel (data not shown). Damaged cells were excluded by 7-AAD staining. VCAM-1 staining in combination with other candidate surface markers for cardiomyocytes, was performed using the following pre-titrated antibodies: rat anti VCAM-1 (10μg/ml, BD Biosciences), mouse anti-rat PE (2μg/ml, BD Biosciences), rat anti PECAM-1-APC (0.5μg/ml, BD Biosciences), or isotype controls rat-IgG2a and rat-IgG2a -APC at corresponding concentrations (BD Biosciences), anti-mouse CD172a (2.5μg/ml SIRP alpha) PerCP-eFluor® 710 (2.5μg/ml eBioscience), goat anti ALCAM (1μg/ml, R&D Systems), Goat IgG (1μg/ml R&D Systems) and anti-goat Alexa Fluor 488 (0.8μg/ml Invitrogen).

**Quantitative PCR**

The following specific primers were used for cDNA amplification:

*α-MHC*: 5´ggtccgggagctggagaatga 3´(F) 5´cttggcccgcagcttgttgac 3´(R)

*β-MHC*: 5´ gtatgaggagtcgcagtcaga 3´(F) 5´gctggatctccatctcgttga 3´(R)

*BNP*: 5´cagctcttgaaggaccaagg 3´(F) 5´agacccaggcagagtcagaa 3´(R)

*CD45:* 5´caaacagagcctcagcctaca 3´(F) 5´ctggacggacacagttagcat 3´(R)

*c-KIT*: 5´acaagaggagatccgcaaga 3´(F) 5´agcaaatcatccaggtccag 3´(R)

*Flk-1*: 5´ccaagctcagcacacagaaa 3´(F) 5´cctgggaatggtgagtgttt 3´(R)

*GAPDH*: 5´gtgctgagtatgtcgtggagt 3´(F) 5´gatggcatggactgtggtcat 3´(R)

*Isl-1*: 5´gccactatttgccacctagc 3´(F) 5´cacagtgaaatggtggttgg 3´(R)

*MLC-2v*: 5´gagctccaacgtgttctccat 3´(F) 5´gccagagccaagacttcctgt 3´(R)

*DDR2*: 5´atctggctttggtggcatac 3´(F) 5´caccaacaaaagtcccgttc 3´(R)

*Vimentin*: 5´tgcacgatgaagagatccag 3´(F) 5´tcttccatctcacgcatctg 3´(R)

*VE-Cad*: 5´tgacagaggccaattcttcc 3´(F) 5´ttactggcaccacatccttg 3´(R)

*Endoglin*: 5´atcatcagtttcccgtcagg 3´(F) 5´tggatgagttccaccatgtc 3´(R)

**Electrophysiology**

Action potential (AP) recordings were obtained in the current-clamp mode of the whole-cell configuration as previously reported^13^. The pipette solution contained (in mmol/L): 50 KCl, 1 MgCl_2_, 3 Mg-ATP, 10 EGTA, 80 Kaspartate and 10 HEPES (pH 7.4; KOH). The extracellular solution was (in mmol/L): 140 NaCl, 5.4 KCl, 1.8 CaCl_2_, 2 MgCl_2_, 10 HEPES, 10 glucose (pH 7.4; NaOH). Muscarinic, β-adrenergic, Adenosine effects on cardiomyocytes were calculated in terms of percentage of variation with respect to normal solution application. Ramps were recorded in voltage-clamp mode of the whole-cell configuration by application of pulses from -100 to -50 in 75 ms and current voltage relationships were constructed. The results are displayed as mean ± SE for *n* cells. The statistical significance of mean values was determined with the paired Student´s t test for the effects of ISO, CCH and ADO on spontaneous beating rates in the same cells and with the unpaired Student´s t test in regard to MDP and APD90. P values <0.05 were considered as statistically significant.

**Ca^2+^ imaging:**

Embryonic cardiomyocytes purified by FACS were re-plated for three days. For imaging experiments cells were loaded with the Ca^2+^ indicator Fura 2-AM (2µM) for 10 minutes at room temperature and transferred to a custom built recording chamber. The bath solution contained (in mM): 140 NaCl, 5.4 KCl, 1 MgCl_2_, 1.8 CaCl_2_, 10 HEPES and 10 glucose.

Ca^2+^ imaging was performed using monochromic excitation light (340, 380 nm) generated by a computer-controlled monochromator. Emitted light was collected through a 470-nm long pass filter using a cooled digital CCD camera (TILL Photonics, Planegg, Germany) coupled with an inverted microscope (Axiovert 200M, Zeiss Microimaging GmbH, Goettingen, Germany). Tetrodotoxin and caffeine were directly added to the bath solution. The emission data were analyzed using the Vision software package (TILL Vision 4.0, Photonics Planegg, Germany). Results are displayed as 340nm/380nm ratios after background subtraction. A response was considered an increase of the 340/380 ratio by more than 10%.

**PKH67 cell membrane labeling assay**

FACS-isolated cardiomyocytes (200.000) were labeled with PKH67 using the PKH67 fluorescent cell linker kit (Sigma-Aldrich) according to the manufacturer. As a control, irradiated embryonic fibroblasts were labeled in parallel. After labeling, stained cells and unstained controls were analyzed by Flow Cytometry. The cells were then cultured for 6 days and analyzed again by Flow Cytometry, using the same settings. The stained cardiac cells were seeded onto unstained irradiated embryonic fibroblasts and the stained irradiated embryonic fibroblasts were grown alone. Unstained irradiated embryonic fibroblasts were grown in parallel to serve as a negative control.
