# Supplementary figures and images for: FACS-Based Isolation, Propagation and Characterization of Mouse Embryonic Cardiomyocytes Based on VCAM-1 Surface Marker Expression
Source: PLoS One. 2013 Dec 30;8(12):e82403. doi: 10.1371/journal.pone.0082403 (PMC3875414; doi:10.1371/journal.pone.0082403)

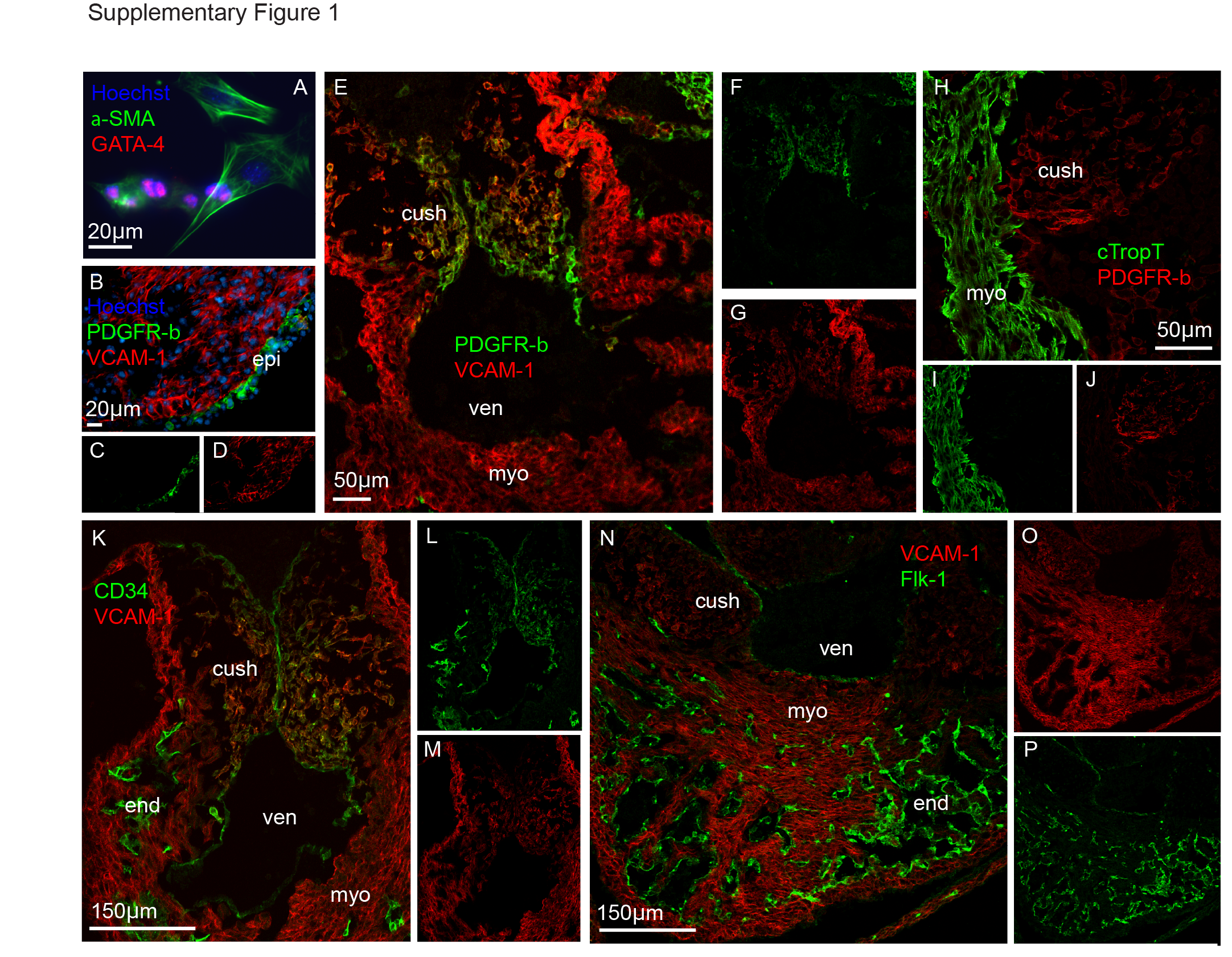

Supplement: Figure S1 — Immuno-localization of VCAM-1 expressing non-cardiac mesenchymal cells. Representative image of a small fraction VCAM-1+ sorted cells expressing α-SMA but not the cardiac marker GATA-4 (A). Immunofluorescence staining of embryonic tissue section showing expression of PDGFR-β in the epicardium, whereas VCAM-1 is expressed by the underlying myocardium (B–D). Optical sections showing co-localization of PDGFR-β with VCAM-1 in mesenchymal cells of the AV-canal cushion (E–G) but not with the surrounding cTropT-positive myocardium (H–J). Optical sections showing co-localization of CD34 with VCAM-1 in mesenchymal cells of the AV-canal cushion but not in endocardial or endothelial cells (K–M). Optical sections demonstrating no co-localization of Flk-1 with VCAM-1 in either myocardium, endocardium, or cushion cells (N–P). Abbreviations: (cush) AV canal cushion (end) endocardium (myo) myocardium (ven) ventricle. Tissue sections: E10.5 (B–M); E11.5 (N–P). (TIF) [file pone.0082403.s002.tif]

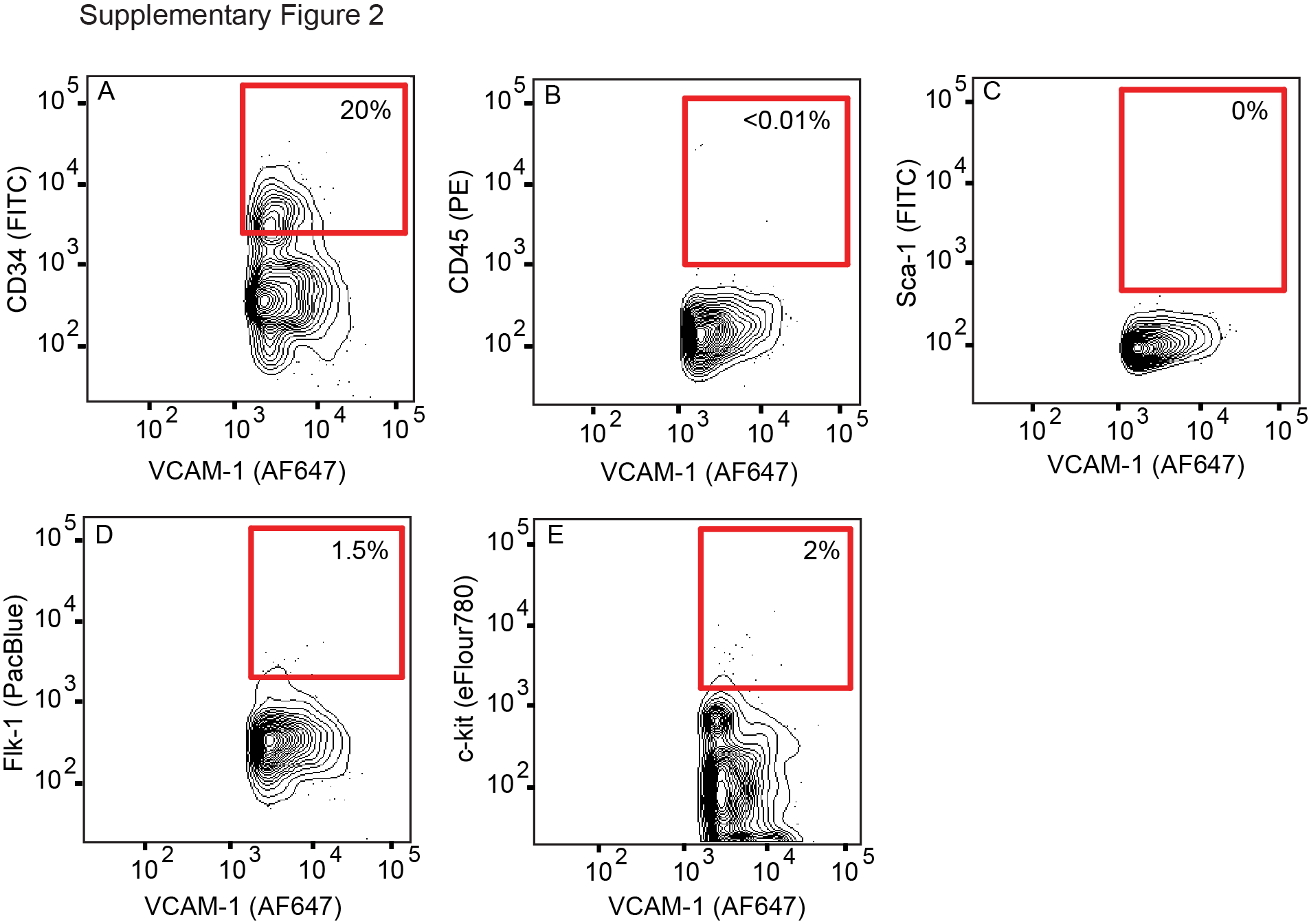

Supplement: Figure S2 — FACS analysis of progenitor and stem cell markers in combination with VCAM-1. Within the VCAM-1 positive population, 20% of the cells where CD34+ (A). No co-expressionwith the common leukocyte antigen CD45 (B) or the hematopoietic stem cell marker Sca-1(C) was detected. A very low fraction of VCAM-1+ cells expressed the progenitor markers Flk-1 or c-kit (D, E). (TIF) [file pone.0082403.s003.tif]

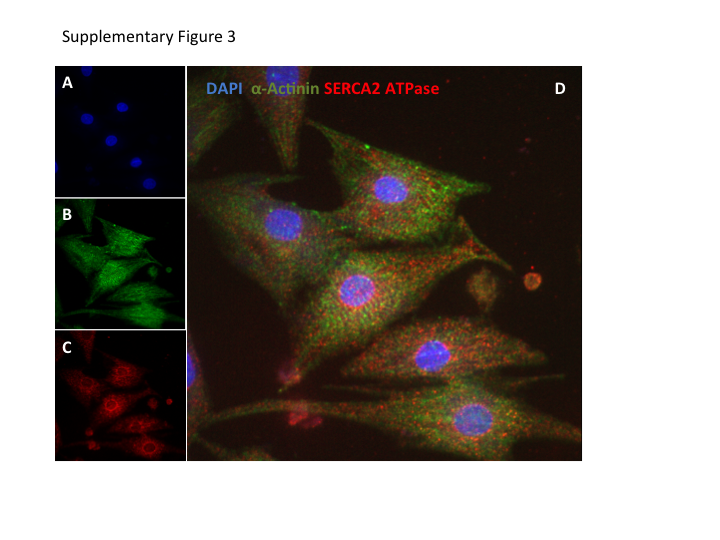

Supplement: Figure S3 — Co-localization between calcium proteins and α-actinin on VCAM-1+/PECAM-1− cells. Immunofluorescence staining on FACS-isolated and cultured cells. Nuclear stain with DAPI (A), α-actinin (B) and SERCA2 ATPase (C) with all channels combined (D). (TIF) [file pone.0082403.s004.tif]

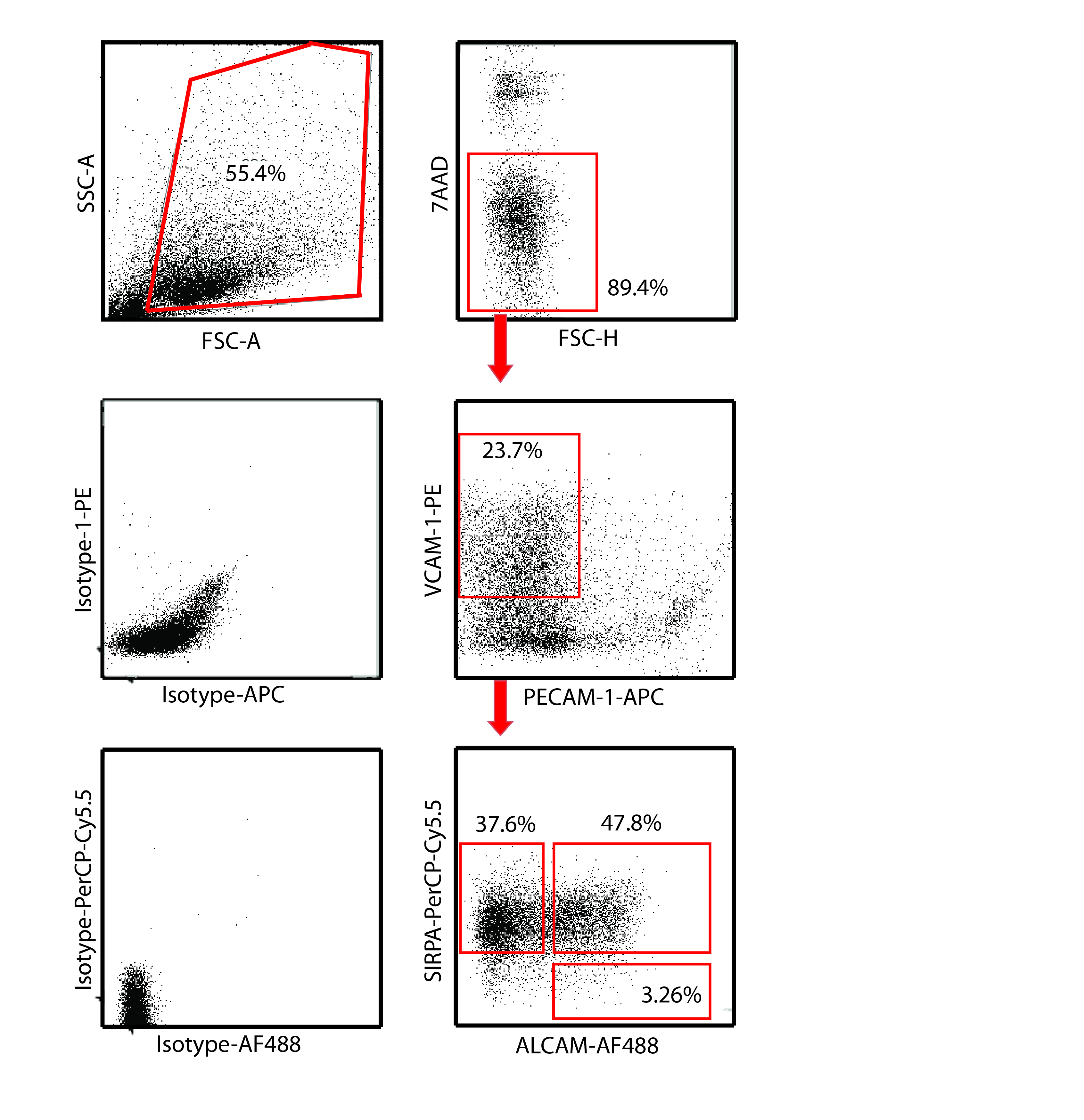

Supplement: Figure S4 — Flow Cytometry analysis of the VCAM-1+/PECAM-1− population and co-localization of SIRPA and ALCAM. FSC-A and SSC-A gating of cells isolated from the heart (A). 89.4% of the cells were viable as assessed by 7-AAD (B). Isotype controls were performed for each antibody used (C, E). The VCAM-1+/PECAM-1− population (D) is to a high degree (85.4%) positive for SIRPA (F) but to a lesser degree ALCAM+ (51.1%) (F). (TIF) [file pone.0082403.s005.tif]

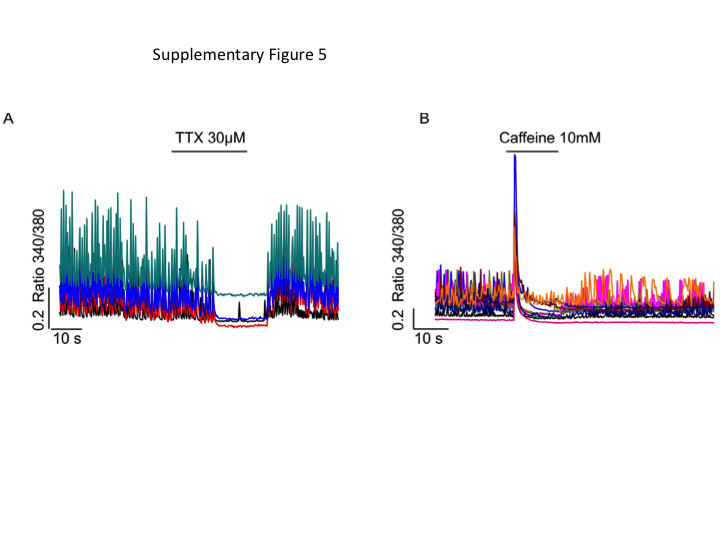

Supplement: Figure S5 — Ca2+ imaging of PECAM− VCAM+ embryonic cardiomyocytes. Spontaneously beating cardiomyocytes displayed regular [Ca2+]i transients, which could be stopped by applying TTX (30 µM) (100%, n = 19), a blocker of voltage activated Na+ channels (A). When applying caffeine (10 mM), most of the cells (88.7%, n = 44) showed a large [Ca2+]i transient (B) due to the emptying of the sarcoplasmatic reticulum stores, followed by a reduced amplitude of the [Ca2+]i transients until refilling of the stores during wash-out. (TIF) [file pone.0082403.s006.tif]
