# Supplementary material for: FACS-Based Isolation, Propagation and Characterization of Mouse Embryonic Cardiomyocytes Based on VCAM-1 Surface Marker Expression
Source: PLoS One. 2013 Dec 30;8(12):e82403. doi: 10.1371/journal.pone.0082403 (PMC3875414; doi:10.1371/journal.pone.0082403)
Supplement: Table S1 — FACS statistics of VCAM-1+ PECAM− cardiomyocytes. (DOC) [file pone.0082403.s009.doc]

**Table S1. FACS statistics of VCAM-1^+^ PECAM^-^ cardiomyocytes**

|  | E9.5-10.5 | E9.5-11.5 | E10.5-11-5 | E11.5-12.5 |
| --- | --- | --- | --- | --- |
| Cell yield/heart^*^ | **6888** | **7253** | **8714** ± **2212** | **5739** ± **5315** |
| Cell viability  (before FACS)^§^ | **95,0** | **95,5** | **96,3** ± **2,1** | **96,7** ± **3,2** |
| Cell viability  (after FACS)^†^ | **95,0** | **97,5** | **96,8** ± **3,5** | **98,3** ± **1,2** |
| Gated cells^#^ | **18,5** | **18,5** | **19,3** ± **2,7** | **9,7** ± **5,5** |
| Purity^‡^ | **85,5** | **94** | **92,8** ± **5,5** | **79,3** ± **2,1** |
| Sorts | **2** | **2** | **6** | **3** |

^*^Average number of cells retrieved from one heart ± standard deviation

^§^ Fraction (%) of viable cells at FACS-isolation ± standard deviation

^†^Fraction (%) of viable cardiomyocytes (100-200 cells) after FACS-isolation ± standard deviation

^#^Sorted fraction VCAM-1^+^ PECAM^-^ (%) from live singlet cells ± standard deviation

^‡^Purity (%) determined by reanalysis of sorted cells (100-200 cells) within the same gate ± standard deviation
