# Supplementary material for: FACS-Based Isolation, Propagation and Characterization of Mouse Embryonic Cardiomyocytes Based on VCAM-1 Surface Marker Expression
Source: PLoS One. 2013 Dec 30;8(12):e82403. doi: 10.1371/journal.pone.0082403 (PMC3875414; doi:10.1371/journal.pone.0082403)
Supplement: Table S2 — Quantitative analysis of FACS-isolated VCAM-1+ PECAM− cardiomyocytes. (DOCX) [file pone.0082403.s010.docx]

**Table S2. Quantitative analysis of FACS-isolated VCAM-1^+^ PECAM^-^ cardiomyocytes**

|  | Fraction | Sorts | Age |
| --- | --- | --- | --- |
| Purity, by cTropT FCM^*^ | 98.3 ± 0.5 % | **4** | **E9.5-11.5** |
| Purity, by cTropT IF^§^ | 96,7 % | **2** | **E10.5-11.5** |
| α-SMA expression^†^ | 100 % | **1** | **E10.5-11.5** |
| GATA-4 expression^†^ | 80 % | **1** | **E10.5-11.5** |
| Nkx2.5 expression^†^ | 85 % | **1** | **E10.5-11.5** |
| Cell proliferation (BrdU) ^#^ | 84 % | **1** | **E11.5** |
| Cell viability‡ | 94,3 ± 1,2 % | **3** | **E10.5-11.5** |

^*^Purity determined by cTropT staining followed by FACS analysis ± standard deviation. 1 sort included E9.5-10.5 embryos and three sorts included E10.5-11.5 embryos.

^§^Purity determined by cTropT immufluorescence staining of plated cells on gelatin

^†^Expression of embryonic cardiac markers, determined by immunofluorescense staining of plated cells on gelatin

^#^Fraction of cultured cardiomyocytes with BrdU-incorporation after 24h

‡Fraction (%) of viable cardiomyocytes (50.000 cells) after FACS-isolation ± standard deviation
